# Prolongation of Postoperative Drainage Time in Indocyanine Green Lymphography as a Potential Marker for Lymphedema Development—A Prospective Pilot Study

**DOI:** 10.3390/jcm15093460

**Published:** 2026-05-01

**Authors:** Karolina Anuszkiewicz, Marcin Ekman, Mateusz Drozd, Kamil Drucis, Jerzy Jankau

**Affiliations:** 1 Division of the Plastic Surgery, Medical University of Gdansk, Mariana Smoluchowskiego 17, 80-214 Gdansk, Poland; 2 Department of Oncological, Transplant and General Surgery, Medical University of Gdansk, Mariana Smoluchowskiego 17, 80-214 Gdansk, Poland

**Keywords:** lymphography, indocyanine (ICG) lymphography, lymphatic drainage, lymphedema, breast cancer

## Abstract

**Objectives**: Lymphedema (LE) is a debilitating complication in breast cancer patients, typically identified through clinical symptoms and volume-based diagnostics. As early diagnosis is crucial for favorable outcomes of microsurgical procedures, a more sensitive tool for LE assessment is required. The primary aim of this study was to evaluate whether a prolongation in postoperative indocyanine (ICG) lymphography drainage time, relative to preoperative baseline values, serves as a predictor of future LE development. **Methods**: A total of 41 women undergoing axillary lymph node dissection received ICG lymphography preoperatively and four weeks postoperatively. Drainage time (the duration for ICG to reach the axilla) was recorded. Clinical LE was defined as a >10% limb volume difference 12 months post-surgery, while subjective LE (sLE) was assessed via the Lymphedema Life Impact Score. **Results**: LE developed in 19.5% of patients. Patients who developed LE exhibited significantly higher mean drainage prolongation compared to those who did not (335 s vs. 40 s; *p* = 0.004). ROC analysis identified an optimal threshold of 119 s for predicting LE, yielding 100% sensitivity and 84.85% specificity (AUC = 0.96). sLE was reported by 48.8% of patients. Their drainage prolongation was significantly greater than in the sLE group (188 s vs. 13 s; *p* = 0.03). **Conclusions**: Preliminary findings suggest postoperative prolongation of ICG drainage time may serve as a potential predictor of future LE. In our cohort, a 119 s delay at four weeks post-operation was associated with LE at 12 months. While these results are promising, further research in larger, more diverse populations is required to validate these thresholds for clinical utility.

## 1. Introduction

Lymphedema (LE) remains one of the most debilitating long-term complications following breast cancer surgery, particularly in patients undergoing axillary lymph node dissection (ALND) [1]. Despite the increasing adoption of sentinel lymph node biopsy (SLNB) and de-escalating trends observed in complete axilla dissection, ALND is still necessary for many patients with confirmed nodal involvement, carrying a 13–30% risk of secondary LE [1,2]. The condition is characterized by a progressive accumulation of protein-rich interstitial fluid, leading to chronic inflammation, tissue fibrosis, and a significant decrease in the patient’s quality of life [3]. Early diagnosis of lymphatic impairment is crucial, as physiological microsurgical interventions demonstrate the highest efficacy when performed during the subclinical or early stages of the disease [4]. However, traditional diagnostic methods, such as limb volume measurements based on circumference, often detect LE only after significant and sometimes irreversible tissue changes have occurred. Indocyanine green (ICG) lymphography has emerged as a highly sensitive tool for visualizing superficial lymphatic architecture and kinetics [5]. While many studies focus on qualitative patterns, such as dermal backflow, the quantitative assessment of lymphatic drainage time offers a more objective measure of lymphatic efficiency [6]. Identifying a reliable physiological marker in the early postoperative period could allow for the selection of high-risk patients who would benefit most from early intervention or prophylactic strategies. The primary aim of this study was to evaluate whether a prolongation in early postoperative ICG drainage time, relative to preoperative baseline values, serves as a predictor of LE development after 12 months post-operation. Additionally, we sought to establish a specific postoperative cut-off threshold to be utilized as a predictive tool for early LE detection.

## 2. Materials and Methods

### 2.1. Study Design and Population

The study was designed as a prospective, observational cohort study including women with unilateral breast cancer who qualified for ALND as part of their treatment. The recruitment period spanned from January 2024 to December 2024. Qualification for ALND was performed in accordance with the Polish Society of Medical Oncology Guidelines which align with the 2023 European Society of Medical Oncology (ESMO) Recommendations. Scenarios included cN0 patients with more than two metastatic lymph nodes in SLNB, and cN2–cN3 patients after neoadjuvant therapy (NAT). In cases of cN1 patients post-NAT, the necessity of ALND was determined through individual assessment by a multidisciplinary team, including a medical oncologist, radiation oncologist, radiologist, and surgical oncologist. Routinely, ALND was performed on level I–II axillary lymph nodes, and on level III only if clinically indicated.

Exclusion criteria included: breast cancer or axillary lymph node surgery on contralateral site in the past, presence of lymphedema, age < 18 years, presence of distal metastases, hyperthyroidism (a contraindication for ICG), language barriers, and a lack of informed consent. The study was approved by the institutional bioethical committee of the Medical University of Gdansk, and all participants provided written informed consent.

### 2.2. Data Collection

Patient demographic and clinical data were collected, including age, comorbidities, body mass index (BMI), and site of the neoplasm. Additional data included receipt of NAT (chemotherapy or hormone therapy), type of breast surgery, history of previous SLNB, and T and N features in pathological TNM classification according to the Union for International Cancer Control (UICC) TNM Classification of Malignant Tumors, 8th Edition [7]. Postoperative data collected included neoplasm molecular subtype, the number of lymph nodes dissected, presence of radiotherapy to the axillary area, chemotherapy, participation in physiotherapy according to our breast center protocol (including 10 individual one-hour meetings with a physiotherapist two weeks after the surgery), and the daily use of compression garments. Neoplasm molecular subtypes were defined based on immunohistochemical assessment of estrogen receptor (ER), progesterone receptor (PR), HER2 status, and Ki-67 proliferation index, in accordance with the St. Gallen International Expert Consensus recommendations [8]. HER2-enriched subtypes were defined as ER-negative and PR-negative tumors with HER2 overexpression or amplification.

### 2.3. ICG Lymphography

**Figure 1:**
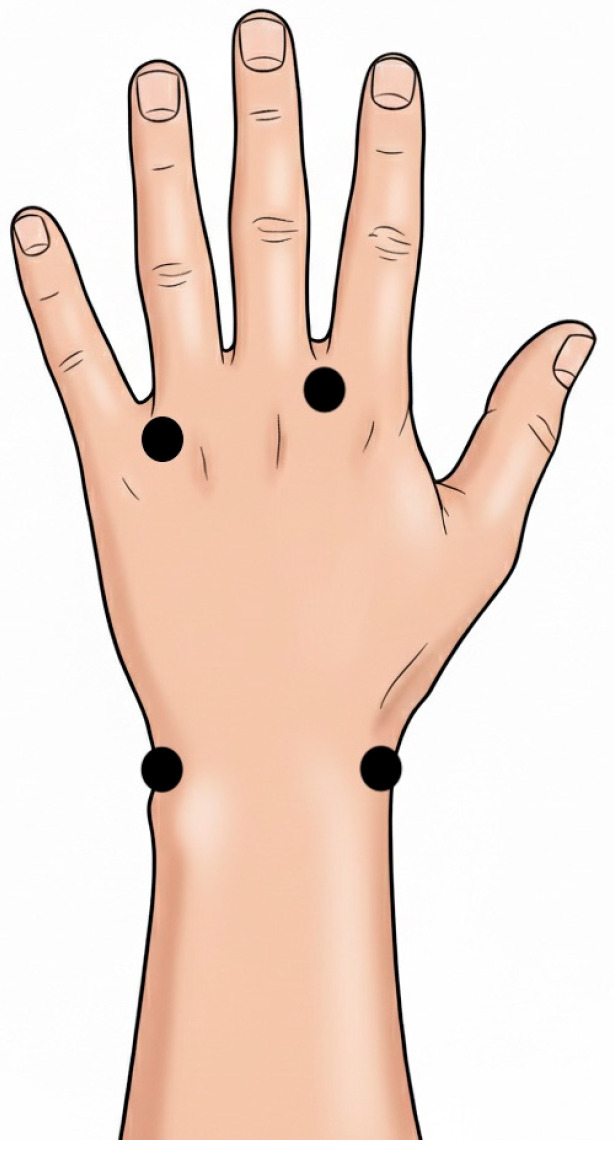
Points of ICG injections. Each black dot refers to one injection point.

ICG lymphography was performed twice for each patient, one day before the ALND procedure and 4 weeks after. To minimize operator bias, all procedures were performed by the first author. Each patient was maintained in a supine position for at least 10 min. Injection sites were first anesthetized subcutaneously with a 1% solution of lidocaine. As there is no standard protocol for the dosage of ICG used in lymphography, the dosage was adapted from previous studies and consisted of 2.5 mg of ICG (Verdye^®^, Diagnostic Green Limited, Athlone Buisiness&Technology Park, Garrycastle, Athlone, Co. Westmeath, Irleand, 25 mg) diluted in 1 mL of aqua per injection [9]. Subcutaneous injections were administered in equal doses (0.25 mL each) at four points: in the second and fourth interdigital webspaces, and at the radial and ulnar areas of the wrist. Schematic visualization of injection points is presented in Figure 1. Patients were instructed to remain still in a supine position and make no movement or muscle tensions with the upper limb for the duration of the test. The limb was abducted at 90 degrees while resting along the side of the torso. After the final injection, a timer was started. ICG lymphography was captured using a Medtronic^®^ ICG camera (EleVision™ IR Platform, Medronic, Minneapolis, MN, USA, 805 nm wavelength, type of visualization—lymphatic vessels), and the time (in seconds) required for the ICG to reach the axillary region (corresponding to the armpit in clinical examination) was recorded for each patient.

### 2.4. Lymphedema Assessment

**Figure 2:**
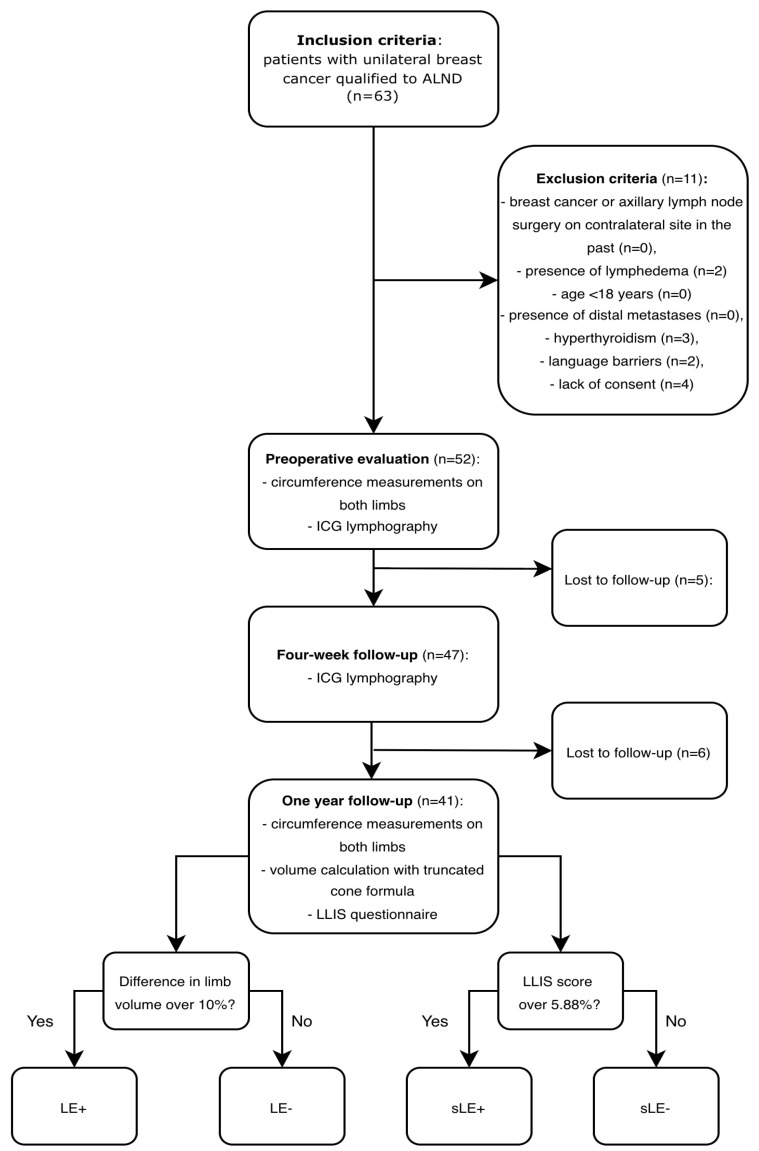
Study protocol. In final cohorts, patients were divided into lymphedema positive (LE+) and negative (LE−) groups according to the presence of 10% difference in limb volumes. Independently, each patient was categorized as subjective lymphedema positive (sLE+) or negative (sLE−) based on their LLIS questionnaire score.

Preoperative and postoperative measurements of both upper limbs were collected for each patient by a single physician (blinded to the result of ICG lymphography drainage time). Preoperative evaluation was made one day before the surgery, while postoperative assessment was conducted 12 months after the ALND procedure. Patients who did not attend the follow-up meeting after one year were excluded. Circumference measurements were noted in centimeters at 4 cm intervals from the wrist to the axilla. Limb volume was estimated using the truncated cone formula. A difference of more than 10% in postoperative volumes between the upper limbs of a single patient was defined as the presence of LE [10]. Based on this volume difference, patients were classified as lymphedema positive (LE+) or lymphedema negative (LE−). Additionally, at the 12-month follow-up meeting, each patient completed the Lymphedema Life Impact Score (LLIS) questionnaire. According to the questionnaire, a quality of life impairment of >5.88% is sufficient for an LE diagnosis, while impairment over 30% refers to a moderate LE diagnosis [11]. Based on quality of life impairment scores, patients were classified as subjective lymphedema positive (sLE+) or subjective lymphedema negative (sLE−). The study protocol is presented in Figure 2.

### 2.5. Statistical Analysis

Statistical analysis was performed using Stata software (version 19, StataCorp, College Station, TX, USA). Continuous variables, such as age, BMI, the number of lymph nodes removed, volume, and drainage time were presented as means with standard deviations (SDs) and ranges (min–max). The normality of data distribution was assessed using the Shapiro–Wilk test. To evaluate the differences between the two groups (preoperatively vs. postoperatively; LE+ vs. LE−; sLE vs. sLE−), unpaired or paired Student’s *t*-tests were employed for normally distributed continuous variables. For variables that did not meet the criteria for normal distribution, the Mann–Whitney U test was used. Categorical variables, including the operated side, type of surgery, previous SLNB, neoplasm type, NAT, radiotherapy status, postoperative chemotherapy, physiotherapy, and compression garment usage were expressed as frequencies. Comparisons between groups for these variables were performed using Pearson’s chi-squared test or Fisher’s exact test, where appropriate (for cell counts below five). A *p*-value of <0.05 was considered statistically significant.

## 3. Results

### 3.1. Patient Characteristics

Forty-one women were included in the study. Information about patient demographics and clinical data are presented in Table 1.

### 3.2. Lymphedema Evaluation

The mean postoperative limb volume on the affected side was 1913 mL (SD 404; 1303–2839), compared to 1808 mL (SD 367; 1111–2681) on the contralateral side. Eight patients (19.5%) met the criteria for clinical lymphedema (LE+) with >10% difference in postoperative volume between the limbs. Statistical analysis revealed significant association between presence of LE and NAT. Statistical analysis between patients’ characteristics and presence of clinical lymphedema is presented in Table 1.

The mean LLIS was 12 points (SD 14; 0–51). A score exceeding the 5.88% threshold (4 points) was observed in 20 patients (48.8%), who were subsequently assigned to the subjective lymphedema (sLE+) group. Among them, eight patients scored over 30%, indicating moderate impairment in quality of life. No statistical association was found between sLE and any patient characteristic.

Mean limb volumes preoperatively and postoperatively for each group are presented in Table 2.

### 3.3. Drainage Time

The mean preoperative ICG drainage time for the entire cohort was 636 s (SD 204; 310–1103), corresponding to 10 min 37 s. No significant difference in preoperative drainage times existed between the groups. The mean postoperative drainage time increased to 734 s (SD 263; 269–1324), which corresponds to 12 min 14 s. Increase in drainage time postoperatively was statistically significant for the entire cohort (*p* = 0.002), and for each group (LE+, LE−, sLE+). Increase in drainage time in sLE− did not achieve statistical significance. All mean drainage times with SD and 95% confidence intervals are listed in Table 3.

**Figure 3:**
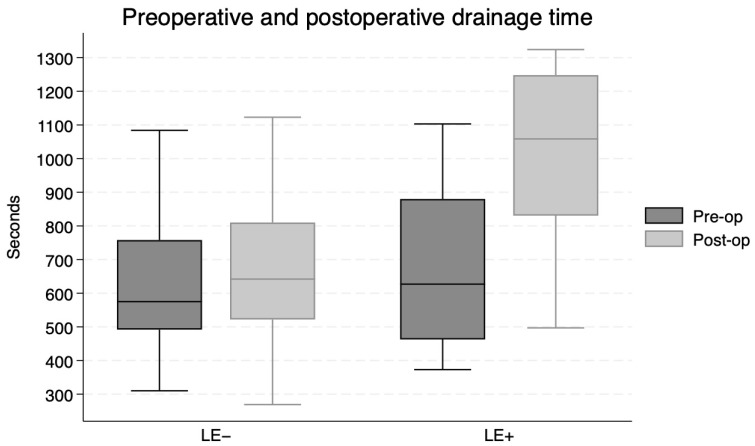
Drainage time preoperatively and postoperatively for lymphedema negative and positive patients.

**Figure 4:**
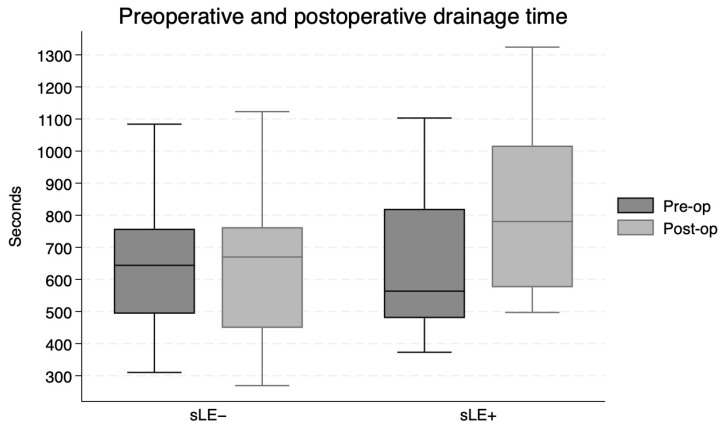
Drainage time preoperatively and postoperatively for subjective lymphedema negative and positive patients.

Prolongation of the drainage time was greater in the LE+ group compared to the LE− group (*p* = 0.004; paired *t*-test), as for the sLE+ group compared to the sLE− group (*p* = 0.03; paired *t*-test). Pre and postoperative drainage times for each group are presented in Figure 3 and Figure 4.

ROC analysis identified an optimal cut-off point for drainage prolongation at 119 s to predict clinical LE (AUC = 0.96, sensitivity 100%, specificity 84.85%). For subjective lymphedema (sLE), the diagnostic threshold was lower, at 69 s of prolongation (AUC = 0.86, sensitivity 85%, specificity 95%).

## 4. Discussion

The results of this study suggest that the prolongation of drainage time in postoperative ICG lymphography, compared to preoperative baseline values, may serve as a potential predictor of future LE development. The impairment of the lymphatic pump, manifested as its slowing, was observed across the entire cohort postoperatively, although the magnitude of this effect determined the symptoms. The four-week timing for the postoperative examination was selected based on clinical consensus at our Breast Center, as this period typically allows for the resolution of postoperative swelling, removal of the external drainage and wound healing [12]. Moreover, this approach facilitates the early identification of patients with subclinical lymphatic impairment before additional treatment modalities, such as radiotherapy, potentially further compromise the lymphatic architecture. One month after the procedure, the remodeling process of lymphatic vessels should be stable, which may allow for an accurate assessment of current lymphatic sufficiency [13,14,15]. ALND causes mechanical disruption of the lymphatic vessels. Disruption induces lymph stasis, which leads to a slowing of drainage, and consequently increases pressure in the distal vessels, which naturally may lead to dermal backflow [16]. In further steps, remodeling starts with lymphangiogenesis and initiation of alternative drainage routes [17,18]. The effectiveness of those processes may expand lymphatic reserve capacity and limit LE progression and severity [18]. As the severity of prolongation differed among the LE+, LE− and sLE+ groups, it may suggest that in some cases remodeling processes are insufficient, which leads to LE progression. In this context, the severity of drainage time prolongation may be considered as a marker for assessing the efficiency of the reparative processes and reflect changes in the pathophysiological state. An additional observation supporting this hypothesis is that the group of patients without subjective symptoms (sLE−) was the only cohort that did not exhibit a significant prolongation in postoperative time.

ICG lymphography gives the ability to visualize real-time lymphatic flow, providing information about lymph drainage physiology. Usually, it is considered as a static imaging technique, analyzing drainage patterns [19]. Our study introduces a novel approach by analyzing quantitative data—time. Under standardized conditions, this approach may be less susceptible to operator bias, more precise, and—as a continuous variable—provide more flexibility in detecting subtle changes. Yamamoto et al. were among the first to evaluate broadly ICG lymphography as dynamic imaging [19]. They demonstrated that dynamic ICG drainage time increases with each stage of LE [9]. Moreover, prolongation was seen in stage 0 patients according to the ISL scale who lacked clinically visible symptoms. In many studies, ICG lymphography was assessed as the most sensitive tool for the visualization of the impairment before symptoms become clinically apparent or felt by the patient [5,20,21,22]. This supports our findings, as in our study prolongation was observed months before the onset of LE. We acknowledge that dynamic changes are sensitive to the patient’s position, movement, and potential dosage of ICG or injection sites and depth [23]. To overcome these limitations, all participants were asked to remain still, with no movement or muscle tension in the examined limb. Furthermore, the dosage and injection protocols were kept identical for each patient, and all examinations were performed by the same researcher to ensure consistency. It remains essential to evaluate inter-operator variability and establish optimal, replicable protocols before routinely integrating this method into clinical practice.

The lack of a universal definition for LE remains a challenge in clinical research. Many studies rely solely on objective measurements, sometimes overlooking the patient’s subjective experience [24]. This leads to reporting varied, sometimes highly divergent, incidence rates of LE [25]. In our cohort, 48.8% of patients reported at least mild impairment in quality of life due to LE-related symptoms—a rate twice as high as the incidence based on clinical criteria alone. A 10% difference in limb volume is a commonly used definition of LE in studies and everyday practice [26]. It is easy to establish and independent of the patient’s weight. For these reasons, it was adopted in our study. Limb volume was assessed by the truncated cone formula, which was evaluated as a reliable tool for volume estimation with high accuracy and correlation compared to results obtained by perometer, 3D scanners or the water displacement technique [27,28]; however, the upper limb does not have a perfect geometrical shape, so the measurements may not be precise. We are aware that choosing different cut-offs may result in different diagnosis rates and drainage times; however, drainage time was also longer for the sLE group, whose diagnosis was based on subjective symptoms alone.

There is an ongoing need for diagnostic tools for early LE identification before the onset of clinical symptoms. Skin thickening visualized via ultrasonography (USG) in the medial upper arm has been identified as a sensitive marker for LE, even in patients with limb volume changes of less than 5% [29]. Infrared thermography may also detect differences in skin temperature, showing a decrease in temperature at the affected site even before volume changes appear [30]. Early identification of at-risk patients is crucial, especially given the current trend of developing LE prophylaxis techniques. We hypothesized that a prolongation of drainage time would precede the appearance of dermal backflow and changes in lymphatic patterns, which are well-established markers of lymphedema development. If so, analysis of prolonged drainage time combined with other techniques may be useful in clinical practice for proper patient selection for preventive procedures at a very early stage, before the onset of dermal backflow and at the reversible stage of the disease. Lymphaticovenous anastomosis (LVA) offers promising results in the treatment of LE as it addresses the underlying cause of the disease rather than merely alleviating symptoms [4]. Several studies have demonstrated the effectiveness of prophylactic LVA performed simultaneously with ALND. Studies evaluating early ICG lymphography findings may contribute to the design of a proper protocol for future clinical practice. We are aware that our investigation should be viewed as a pilot study due to the limited sample size; however, we believe it delivers interesting findings regarding the use of ICG lymphography as a dynamic tool.

This investigation has several limitations. Due to the limited number of patients in the LE+ group, multivariate analysis was not performed as it could not achieve proper statistical power. None of the known LE development risk factors (high BMI, radiotherapy to the axilla) were significantly associated with prolongation of the drainage time, although this may be a consequence of the limited sample size. The only feature associated with LE development was administration of NAT; however, NAT is commonly administered if lymph node metastases are present, which raises the question of whether NAT alone, or the combination of metastases and NAT, increases the risk of lymphedema. Future analysis should focus on changes in drainage time both before and after NAT. The single-center design may limit the generalizability of our findings and utilizing a single operator does not allow for the assessment of inter-observer reliability. Future studies should include larger cohorts to facilitate external validation and inter-operator comparisons. We acknowledge that the results for certain patients may be confounded by individual variability in wound healing and residual postoperative changes. Future studies could consider the benefits of additional examinations at a later time period. Our 12-month follow-up period captures the peak incidence of LE following ALND [31]; however, patients with a later onset of LE may have been missed. All of the above may limit the interpretation of our ROC analysis. While the analysis demonstrated high accuracy for LE prediction, identifying a cut-off threshold of 119 s of prolongation, this specific value may be confounded by the limitations. Nevertheless, we believe the results of this study demonstrate a tendency toward prolonged drainage times, although specific cut-off time must be validated in a larger patient cohort.

In conclusion, early postoperative quantitative ICG lymphography may be considered as a marker of physiological change in lymphatic drainage. In our preliminary observational study, prolongation of postoperative drainage time was reflected in future LE development. In our cohort, a prolongation with a threshold of 119 s at the four-week postoperative examination was associated with LE development after 12 months postoperatively. Severity of prolongation was associated with subjective or clinical symptoms. While these findings bring novel and interesting viewpoints, they should be considered as a pilot study. Further research on a larger population is required to validate thresholds and establish clinical utility.

## Figures and Tables

**Table 1:** Patients’ characteristics distinguished by the presence of lymphedema (LE+ and LE−). Continuous variables are given as mean with SD and range (min–max). Two sample Student *t*-tests were used for comparing continuous variables between LE+ and LE− groups. For categorical variables, the chi-squared test was performed.

Patient Characteristic	General (*n* = 41)	LE+ (*n* = 8)	LE− (*n* = 33)	*p*
Age (years)	55.3 (SD 12.8; 31–81)	57.1 (SD 15.5; 39–78)	54.9 (SD 12.3; 31–81)	0.67
BMI (kg/m^2^)	27 (SD 4.9; 17–39)	27 (4.3; 22–36)	26.8 (SD 5.1; 17–39)	0.7
Site of neoplasm (*n*)	Left (*n* = 17) Right (*n* = 24)	Left (*n* = 4) Right (*n* = 4)	Left (*n* = 13) Right (*n* = 20)	0.7
Number of axillary lymph nodes dissected (*n*)	14 (SD 6; 5–34)	16 (SD 8; 5–27)	14 (SD 6; 5–34)	0.74
Breast surgery type (*n*)	Breast-conserving surgery (*n* = 23), immediate implant- or expander-based reconstruction (*n* = 11), mastectomy (*n* = 7)	Breast-conserving surgery (*n* = 3), immediate implant- or expander-based reconstruction (*n* = 4), mastectomy (*n* = 1)	Breast-conserving surgery (*n* = 20), immediate implant- or expander-based reconstruction (*n* = 7), mastectomy (*n* = 6)	0.3
Previous SLNB (*n*)	Yes (*n* = 14) No (*n* = 27)	Yes (*n* = 3) No (*n* = 5)	Yes (*n* = 22) No (*n* = 11)	0.5
Neoplasm molecular subtypes (*n*)	Luminal A (*n* = 12), Luminal B HER2+ (*n* = 8), Luminal B HER2− (*n* = 15), Triple-Negative (*n* = 2), HER2+ (*n* = 4)	Luminal A (*n* = 3), Luminal B HER2+ (*n* = 3), Luminal B HER2− (*n* = 2), Triple-Negative (*n* = 0), HER2+ (*n* = 0)	Luminal A (*n* = 9), Luminal B HER2+ (*n* = 5), Luminal B HER2− (*n* = 13), Triple-Negative (*n* = 2), HER2+ (*n* = 4)	0.56
Tumor feature in pTNM (*n*)	T0 (*n* = 7) Tis (*n* = 2) T1 (*n* = 16) T2 (*n* = 15) T3 (*n* = 1)	T0 (*n* = 3) Tis (*n* = 0) T1 (*n* = 3) T2 (*n* = 2) T3 (*n* = 0)	T0 (*n* = 4) Tis (*n* = 2) T1 (*n* = 13) T2 (*n* = 13) T3 (*n* = 1)	0.52
Lymph node involvement (pN) (*n*)	N0 (*n* = 12) N1 (*n* = 17) N2 (*n* = 10) N3 (*n* = 2)	N0 (*n* = 3) N1 (*n* = 4) N2 (*n* = 1) N3 (*n* = 0)	N0 (*n* = 0) N1 (*n* = 13) N2 (*n* = 9) N3 (*n* = 2)	0.52
Neoadjuvant chemotherapy (*n*)	Yes (*n* = 28) No (*n* = 13)	Yes (*n* = 8) No (*n* = 0)	Yes (*n* = 20) No (*n* = 13)	*0.03*
Postoperative axilla radiotherapy (*n*)	Yes (*n* = 28) No (*n* = 13)	Yes (*n* = 5) No (*n* = 3)	Yes (*n* = 23) No (*n* = 10)	0.7
Postoperative chemotherapy (*n*)	Yes (*n* = 21) No (*n* = 20)	Yes (*n* = 4) No (*n* = 4)	Yes (*n* = 17) No (*n* = 16)	0.99
Postoperative physiotherapy (*n*)	Yes (*n* = 37) No (*n* = 4)	Yes (*n* = 7) No (*n* = 1)	Yes (*n* = 30) No (*n* = 3)	0.99
Postoperative compression therapy (*n*)	Yes (*n* = 19) No (*n* = 22)	Yes (*n* = 3) No (*n* = 5)	Yes (*n* = 16) No (*n* = 17)	0.7

**Table 2:** Limb volumes preoperatively and postoperatively. All values are in mL with SD and range. Paired Student *t*-tests were performed to assess the difference between neoplasm and contralateral site for each group.

	Preoperative			Postoperative		
	Neoplasm Site	Contralateral	*p*	Neoplasm Site	Contralateral	*p*
General (*n* = 41)	1600 (SD 358; 964–2531)	1583 (SD 378; 1039–2763)	0.49	1914 (SD 400; 1303–2839)	1808 (SD 366; 1111–2681)	0.01
LE+ (*n* = 8)	1575 (SD 299; 1161–2077)	1583 (SD 359; 1113–2236)	0.88	2164 (SD 395; 1594–2839)	1831 (SD 449; 1111–2486)	0.001
LE− (*n* = 33)	1605 (SD 375; 964–2531)	1583 (SD 388; 1039–2763)	0.37	1854 (SD 383; 1303–2687)	1803 (SD 351; 1293–2681)	0.01
sLE+ (*n* = 20)	1643 (SD 358; 964–2531)	1594 (SD 378; 1039–2763)	0.17	1957 (SD 338; 1429–2642)	1789 (SD 335; 1111–2520)	0.001
sLE− (*n* = 21)	1558 (SD 368; 964–2477)	1578 (SD 425; 1039–2763)	0.62	1873 (SD 456; 1303–2839)	1826 (SD 401; 1239–2681)	0.06

**Table 3:** Drainage time preoperatively and postoperatively. All values are in seconds with SD and 95% confidence intervals (95% CI). Mean prolongation is given in seconds and was counted as the difference between the mean postoperative time and the mean preoperative time. Paired Student *t*-tests were performed to assess the difference between preoperative and postoperative times.

	Preoperative	Postoperative	Mean Prolongation	*p*
General (*n* = 41)	636 (SD 204; 95% CI 572–701)	734 (SD 263; 95% CI 651–817)	98	0.002
LE+ (*n* = 8)	676 (SD 255; 95% CI 463–890)	1011 (SD 209; 95% CI 768–1255)	335	0.001
LE− (*n* = 33)	627 (SD 193; 95% CI 558–695)	667 (SD 211; 95% CI 592–741)	40	0.004
sLE+ (*n* = 20)	638 (SD 211; 95% CI 539–737)	824 (SD 279; 95% CI 693–955)	188	0.002
sLE− (*n* = 21)	635 (SD 201; 95% CI 543–727)	648 (SD 221; 95% CI 548–749)	13	0.21

## Data Availability

The original contributions presented in this study are included in the article.
